# Beam-induced redox chemistry in iron oxide nanoparticle dispersions at ESRF–EBS

**DOI:** 10.1107/S1600577522011523

**Published:** 2023-01-13

**Authors:** Sabrina L. J. Thomä, Mirijam Zobel

**Affiliations:** a Institute of Crystallography, RWTH Aachen University, Jägerstraße 17–19, Aachen, 52066 Nordrhein-Westfalen, Germany; ESRF and Université Grenoble Alpes, France

**Keywords:** beam-induced radiolysis, radiation damage on inorganic materials, ESRF–EBS

## Abstract

The storage ring upgrade of the European Synchrotron Radiation Facility makes ESRF–EBS the most brilliant high-energy fourth-generation light source, enabling *in situ* studies with unprecedented time resolution. While radiation damage is commonly associated with degradation of organic matter such as ionic liquids or polymers in the synchrotron beam, this study clearly shows that highly brilliant X-ray beams readily induce structural changes and beam damage in inorganic matter, too. Here, the reduction of Fe^3+^ to Fe^2+^ in iron oxide nanoparticles by radicals in the brilliant ESRF–EBS beam, not observed before the upgrade, is reported. Radicals are created due to radiolysis of an EtOH–H_2_O mixture with low EtOH concentration (∼6 vol%). In light of extended irradiation times during *insitu* experiments in, for example, battery and catalysis research, beam-induced redox chemistry needs to be understood for proper interpretation of *insitu* data.

## Abstract 2

With the increased brilliance at the European Research Facility–Extremely Brilliant Source (ESRF–EBS), a beam-induced reduction of non-stochiometric iron oxide nanoparticles (almost maghemite composition) to magnetite was observed in a mixture of ethanol and water with low ethanol concentration.

## 1. Introduction

During the past few decades the brilliance of modern synchrotron light sources has increased a lot (Raimondi, 2016 ▸) and mankind is constantly producing orders of magnitudes more photons in smaller and smaller cross sections (Bras *et al.*, 2021 ▸). After the Extremely Brilliant Source (EBS) upgrade, the European Synchrotron Radiation Facility (ESRF) now produces an X-ray beam being ∼100 times more brilliant than before (Raimondi, 2016 ▸). Increase in brilliance comes with a vast increase in experimental possibilities, that enable us to capture faster events than ever (Bras *et al.*, 2021 ▸) and to achieve the same time resolution on weakly scattering amorphous samples as before on crystalline ones (Vaughan *et al.*, 2020 ▸). Yet, unprecedented photon flux comes with higher risk of radiation damage (Bras *et al.*, 2021 ▸). In macromolecular crystallography, radiation damage is a big issue that the community has been aware of for many years, constantly working on predicting damage thresholds and developing methods on how to prevent it (Bras *et al.*, 2021 ▸; Fourme *et al.*, 2012 ▸; Garman, 2010 ▸; Holton, 2009 ▸). Further, also in other diffraction (Vaselabadi *et al.*, 2016 ▸; Hopkins & Thorne, 2016 ▸; Neuhold *et al.*, 2012 ▸) as well as imaging (Lai *et al.*, 2013 ▸; Wang *et al.*, 2009 ▸; Beetz & Jacobsen, 2003 ▸) and spectroscopy (Rightor *et al.*, 1997 ▸; Chidambaram *et al.*, 2001 ▸) experiments, X-ray radiation damage was observed on radiation-sensitive, mostly organic, materials and studied for future obviation. On the contrary, inorganic solid materials are not expected to be affected by radiation damage in X-ray scattering experiments, since very high radiation doses [MGy; 1 Grey (Gy) = 1 J kg^−1^] are needed for beam-induced changes like the creation of interstitial-hole Frenkel-pairs, excited electronic states, phase transitions and induced crystallization (Bras *et al.*, 2021 ▸; Tiliks *et al.*, 1991 ▸). Yet, recently, organometal halide perovskites, applied in solar cells, were found to suffer from X-ray induced electronic degradation, namely a decrease of the X-ray beam-induced current (XBIC), even though the elemental composition in X-ray fluorescence (XRF) with common *operando* synchrotron nanoprobe conditions was not found to change (Stuckelberger *et al.*, 2020 ▸). Such a material also exhibited a crystalline lead halide degradation phase, caused by iodine migration from the perovskite, in a scanning nano X-ray diffraction experiment (Ferrer Orri *et al.*, 2022 ▸). Further, radiation damage under X-ray exposure was studied for inorganic materials (metal alloy, oxide and semiconducting) with X-ray photon spectroscopy (Astley *et al.*, 2022 ▸). Changes in the binding energies were identified for all three materials, but due to different reasons, for example thermal expansion of the metal alloy.

Here, we report on beam-induced changes in redox chemistry in iron oxide and associated changes in lattice parameters, which we observed while measuring total scattering on iron oxide nanoparticles (IONPs) dispersed in ethanol–water (EtOH–H_2_O) mixture with low ethanol (∼6 vol%) concentration at ID31 ESRF–EBS with high-energy X-rays (65 keV).

Redox chemistry is at the heart of the working principle of electrochemical and various catalytic processes. The number of *in situ* and *operando* experiments in these fields has risen rapidly because of the unprecedented time-resolution, enabling insight into structural details of, for instance, heterogeneous catalysts dispersed in liquid electrolytes in fuel cells (Chattot *et al.*, 2021 ▸; Martens *et al.*, 2021 ▸). Given the Fe^3+^ → Fe^2+^ beam-induced reduction in IONPs within only 12 s of exposure, what does happen to nanostructured, often multivalent, metal oxides exposed over hours during *operando* experiments?

Many battery materials consist of spinel oxides, thus similar to our IONPs (Choi & Manthiram, 2006 ▸; Koga *et al.*, 2013 ▸; Manthiram, 2020 ▸), and we want to create awareness that charge–discharge processes of batteries could similarly be affected due to radiolysis of the surrounding organics. Further, nanomaterial research and general solvation effects on solid–liquid interfaces may be induced in the future (Roy *et al.*, 2021 ▸; Christensen *et al.*, 2021 ▸; Aalling-Frederiksen *et al.*, 2021 ▸). Besides direct impact of the radicals on the solid state structure, solvated radicals can massively impact chemical reactions, for instance *operando* catalysis experiments. Since materials with excited states behave chemically different, we suppose that increased reaction rates (Bras *et al.*, 2021 ▸) or unforeseen influences of radiolysis products at solid–liquid or liquid–gas interfaces (Le Caër, 2011 ▸; Bras *et al.*, 2021 ▸) cannot be excluded. Hence, reaction kinetics of the experiment may be altered in comparison to without beam.

## 2. Data collection and data treatment

Total scattering measurements were performed at ID15-A at ESRF before the EBS upgrade and at ID31 at ESRF after the EBS upgrade, for IONPs in EtOH–H_2_O mixture with low EtOH concentration, IONPs in water (only ID31) (for information regarding sample preparation see Section S1 of the supporting information) and water, taken in 1 mm Kapton capillaries. Both beamlines are equipped with a PILATUS3 X CdTe 2M detector (253.7 mm × 288.8 mm sensitive area, 172 µm × 172 µm pixel size). For data processing and treatment, we used the following: for masking *Fit2D* and for calibration *pyFAI-calib2* (Ashiotis *et al.*, 2015 ▸), for radial integration *xpdtools* (https://xpdacq.github.io/xpdtools/), for pair distribution function (PDF) processing *PDFgetX3* (Juhás *et al.*, 2013 ▸), for PDF modelling *DiffPy-CMI* (Juhás *et al.*, 2015 ▸) and *PDFgui* (Farrow *et al.*, 2007 ▸), and for fitting of *I*(*Q*) data *IgorPro* by WaveMetrics.

### 2.1. Details on data collection on ID15A – pre-EBS upgrade

Data were taken with an energy of 68 keV (0.1823 Å) and a beam size of about 120 µm × 120 µm [vertical × horizontal (v × h), being a little bit smaller in the vertical] at ID15A before the upgrade. The photon flux on the samples was estimated to be 5 × 10^10^ to 1 × 10^11^ photons s^−1^ (see Section S2 of the supporting information). Ten scans of 25 s each were taken on every sample resulting in a total radiation dose of ∼195–390 kGy (see Section S2 of the supporting information for calculation). NIST chromium(III) oxide standard was used for distance calibration and determination of instrumental resolution (*Q*_damp_ = 0.0181 Å^−1^; *Q*_broad_ = 0.0185 Å^−1^).

### 2.2. Details on data collection on ID31 – after-EBS upgrade

Data were taken with an energy of 65 keV (0.1907 Å) and a beam size of about 100 µm × 300 µm (v × h, ±50 µm) at ID31 after the upgrade. The photon flux on the samples was 1 × 10^14^ photons s^−1^. Ten scans of 6 s each were taken on every sample resulting in a total radiation dose of ∼40000 kGy (see Section S2 of the supporting information for calculation). NIST cerium(IV) oxide standard was used for distance calibration and determination of instrumental resolution (*Q*_damp_ = 0.0159 Å^−1^; *Q*_broad_ = 0.0119 Å^−1^).

## 3. Results and discussion

**Figure 1:**
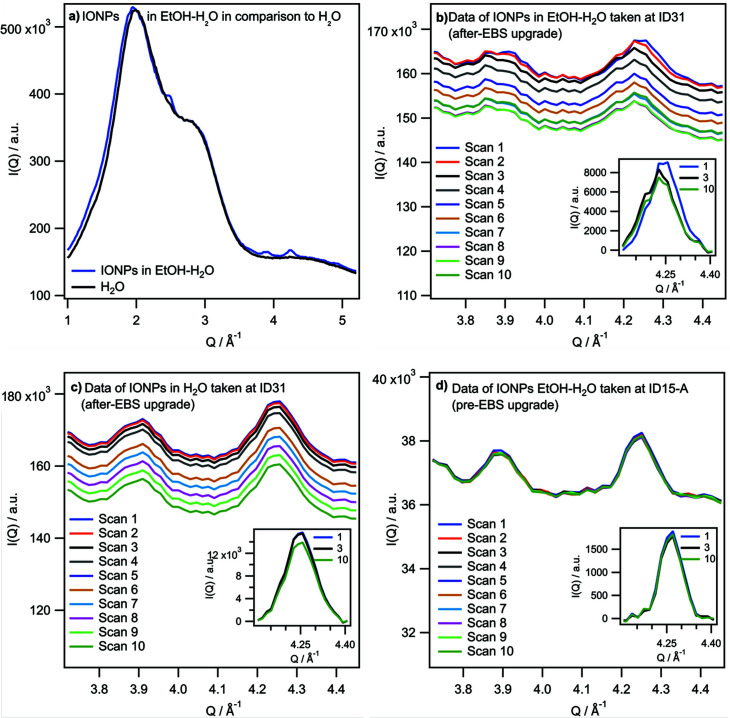
(*a*) *I*(*Q*) data in the *Q*-range of the FSDP for water and IONPs dispersed in EtOH–H_2_O mixture with low EtOH concentration, showing that the FSDP is slightly shifted for the mixture. (*b*) Evolution of *I*(*Q*) scans of IONPs in EtOH–H_2_O mixture with low EtOH concentration taken over time at ID31 after the EBS upgrade between 3.7 and 4.5 Å^−1^ showing that the Bragg reflexes in this region shift to lower *Q*-values after the first scan. The inset shows scans 1, 3 and 10 in the region of one of those Bragg reflexes corrected for the water background to point out the shift. (*c*) *I*(*Q*) scans for the same IONPs just dissolved in pure H_2_O for the same *Q*-range. Scans were taken with the same acquisition time, beam size and with the same flux as data in panel (*b*). No shift can be observed as pointed out in the inset showing the same Bragg reflex corrected for water background as inset of panel (*b*). (*d*) *I*(*Q*) scans of IONPs in EtOH–H_2_O mixture with low EtOH concentration prepared the same way as in panel (*b*) but measured at ID15-A before the EBS upgrade with lower photon flux on the sample. No shift can be observed; see also inset.

Performing total scattering on IONPs of 7 and 15 nm in diameter, dispersed in an EtOH–H_2_O mixture with low EtOH concentration (6 vol%) at ESRF–EBS, we observed shifts of the Bragg peaks over the total time of exposure of 60 s, whereby the biggest shift was observed between the first and second scan between 6 and 12 s. Initially, the IONPs feature an inverse spinel structure with a composition close to maghemite (see Section S3 of the supporting information). Fig. 1 ▸(*a*) shows the diffraction pattern of such an IONP dispersion in the *Q*-range of 1–5 Å^−1^ in comparison with that of water. The diffraction patterns are similar, yet the first sharp diffraction peak (FSDP) of the dispersion is broadened and slightly shifted to lower *Q*-values due to the low amount of EtOH (∼6 vol%; for more information about the EtOH content see Section S4 of the supporting information). The Bragg peaks of the IONPs are visible on top of the broad diffraction from the solvent. In Fig. 1 ▸(*b*) the ten scans taken successively at ID31 after the EBS upgrade are shown in the *Q*-range of the (333) and (440) Bragg reflexes between 3.7 and 4.5 Å^−1^, for one exemplary dispersion of IONPs in EtOH–H_2_O mixture with low EtOH concentration (ID31-1-EtOH–H_2_O). Evidently, both peaks shift to lower *Q*-values after the first scan. This becomes even clearer in the inset which shows the (440) Bragg reflex for scan 1, 3 and 10 only, corrected for the water background. The Bragg peaks of the same IONPs dispersed in solely water (ID31-1-H_2_O) did not shift – see Fig. 1 ▸(*c*). Further, we compared these data with data of samples prepared the same way taken at ID15-A ESRF before the EBS upgrade, thus with lower photon flux per irradiated volume, and consequently resulting in lower total radiation dose despite the smaller beam size and longer exposure time ( *cf*. Section S2 of the supporting information). In this case, too, no peak shift of the IONP Bragg peaks over time could be observed – see Fig. 1 ▸(*d*) (shown for ID15-A-1-EtOH–H_2_O).

We proved the reproducibility of the shift of Bragg peaks in EtOH–H_2_O mixtures after the upgrade (case 1) and its lack in only H_2_O after the upgrade (case 2) and the lack in EtOH–H_2_O mixtures before the upgrade (case 3), for several samples each. In order to quantify the shift and make sure it is bigger than the uncertainty in instrumental resolution, three scans (1, 3 and 10) of two samples of each of the three cases were investigated in more detail. For those scans a Gaussian function was fitted to the (440) reflex in *I*(*Q*) data (see Section S5 of the supporting information). Since a shift to lower *Q*-values is associated with a lattice expansion, this lattice expansion was also confirmed and quantified by PDF fits on the differential-PDFs (d-PDF, IONP dispersion minus water background) (see Section S6 of the supporting information). The results of both evaluations are listed in Table 1 ▸ in comparison with the total applied radiation dose, clearly showing the correlation of radiation dose and Bragg peak shift.

**Figure 2:**
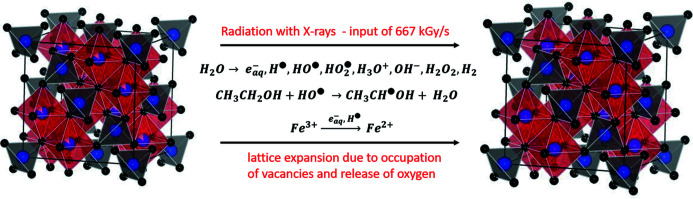
Change of the crystalline structure of the IONPs dispersed in an EtOH–H_2_O mixture with low EtOH concentration upon irradiation with highly brilliant X-rays. Illustrations of the 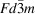 unit cells have been made using *VESTA* (Momma & Izumi, 2011 ▸). Black balls represent oxygen atoms, blue balls represent iron atoms, vacancies are illustrated by partial filling and the red and grey polyhedra illustrate [FeO_6_] octahedral and [FeO_4_] tetrahedral units, respectively. IONPs before radiation with the X-ray beam are non-stochiometric, but mostly containing Fe^3+^ (see Section S3 of the supporting information), crystallized in inverse spinel structure (here described with 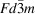 unit cell for simplicity), therefore possessing vacancies on the octahedral sites (left side). Induced by the irradiation with X-rays, both oxidative and reducing radicals are created by radiolysis of water. Due to the presence of EtOH, the oxidative radical 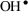 is scavenged, a reducing atmosphere is created and Fe^3+^ is reduced to Fe^2+^, which fills the vacancies and leads to expansion of the crystal lattice due to its bigger ionic radius (unit cell on the right side).

When water is irradiated with ionizing radiation, like radioactive nuclei, beams of charged particles and X-rays with a photon energy >100 eV, radiolysis of water takes place (Le Caër, 2011 ▸; Zhang *et al.*, 2012 ▸). Thereby, many different species are formed, such as hydrated electrons, di­hydrogen and hydrogen peroxide molecules, oxonium and hydroxyl ions, as well as hydroxyl radicals and hydrogen atoms. Hydrated electrons and hydrogen are strong reducing agents, which are known to readily reduce dissolved metal ions to their lower oxidation state, in contrast to the hydroxyl radical, which is oxidative (Le Caër, 2011 ▸). Alcohols are known to be scavengers for the oxidative hydroxyl radical (Simic *et al.*, 1969 ▸; Zhang *et al.*, 2012 ▸; Yamaguchi *et al.*, 2016 ▸). By abstraction of hydrogen from the alcohol, the oxidative hydroxyl radical is scavenged by the alcohol, and thereby organic reducing radicals are evolving (Yamaguchi *et al.*, 2016 ▸; Zhang *et al.*, 2012 ▸). Moreover, upon radiation with X-rays, ethanol can also be oxidized to acetaldehyde via the production of two electrons and two protons (Yamaguchi *et al.*, 2016 ▸). Consequently, in a mixture of water and alcohol a reducing environment for chemical reactions is created upon irradiation with ionizing radiation. For instance, graphene oxide can be reduced to graphene in such a reducing medium with comparable alcohol content (Zhang *et al.*, 2012 ▸). Hence, we are convinced that in our case the non-stochiometric (mostly Fe^3+^containing) IONPs are reduced to magnetite. Magnetite crystallizes in the inverse spinel structure, with O^2−^ anions forming a cubic close packing, Fe^3+^ ions occupying the tetrahedral sites and a 1:1 mixture of Fe^2+^ and Fe^3+^ ions on the octahedral sites (Cervellino *et al.*, 2014 ▸). Magnetite nanoparticles stored in air are fully or partially oxidized to maghemite over time, by creation of vacancies on the octahedral sites accompanied by a lattice contraction (Cervellino *et al.*, 2014 ▸; Sidhu *et al.*, 1977 ▸). In maghemite (γ-Fe_2_O_3_) all of the initial one-third of Fe^2+^ was oxidized to Fe^3+^, and thus the number of vacancies is maximal. These vacancies can either be randomly distributed on the octahedral sites or vacancy ordering can exist, lowering the symmetry of the structure (Cervellino *et al.*, 2014 ▸). In our case in the synchrotron beam, in the reducing medium created upon irradiation with the highly brilliant X-rays, the opposite reaction is taking place: parts of the Fe^3+^ ions are reduced to Fe^2+^ whereby the vacancies are filled again, and the lattice is expanding, due to the bigger ionic radius of Fe^2+^ in comparison with Fe^3+^ (Sidhu *et al.*, 1977 ▸). Since no other iron source besides the IONPs is present, this reduction will most likely be accompanied by the release of oxygen from the crystal structure for providing charge balance. For full conversion from maghemite to magnetite the release would amount to ∼11% of the initial oxygen content of the IONPs. Oxygen present at the IONP surface and in the dispersion could then also be involved in reactions with the evolving radicals. The process is schematically shown in Fig. 2 ▸. This assumption matches the experimental observations very well, since at first the Bragg peaks shift from first to second scan, strongly. Then, an equilibrium state seems to be reached, matching the fact that all vacancies are filled at some point. Further, the radiation dose per second (see Section S2 of the supporting information) in our experiments has been higher than the total radiation doses reported in studies which aimed at synthesizing inorganic nanoparticles via radiolysis by deliberate exposure of metal ion precursor solutions to strong ionizing radiation (Čubová & Čuba, 2020 ▸). We exclude that the expansion is thermal expansion for two reasons: in the case of thermal expansion the IONPs in water only should be likewise influenced considering similar thermal conductivity; further, the increase of the lattice parameter of ∼0.03 Å would be caused at temperatures way above the boiling point of water (Bayer, 1972 ▸; Petric & Ling, 2007 ▸; Levy *et al.*, 2004 ▸), but no evaporation of the solvent is observed in the *I*(*Q*) scans.

## 4. Conclusion

In summary, we have observed beam-induced structural changes in iron oxide nanoparticles created by radiolysis during total scattering experiments on nanoparticle dispersions in an EtOH–H_2_O mixture with low EtOH concentration. The iron oxide nanoparticles were reduced from an almost maghemite (mainly Fe^3+^ containing) composition to magnetite in the highly brilliant synchrotron beam at ID31, ESRF–EBS. This is, to our knowledge, the first showcase of beam-induced damage in inorganic solids after the ESRF upgrade. Given its nature in redox chemistry and evolving radicals in solution, we expect this study to be highly relevant to a range of fields including *in situ* studies in catalysis and energy applications such as fuel cell or battery research. Time-dependent sample-specific changes need to be questioned in light of possibly radiolysis-induced redox chemistry.

## 5. Related literature

The following references, not cited in the main body of the paper, have been cited in the supporting information: Bondaz *et al.* (2020 ▸); Caruntu *et al.* (2004 ▸); Cooper *et al.* (2020 ▸); Greaves (1983 ▸); Qu *et al.* (2011 ▸); Thomä *et al.* (2019 ▸).

## Supplementary Material

Sections S1 to S6, including Tables S1 to S5 and Figures S1 to S4. DOI: 10.1107/S1600577522011523/fv5157sup1.pdf

## Figures and Tables

**Table 1:** Shifts in the (440) reflex and expansion of lattice parameter (from d-PDF fit) in comparison with estimated radiation dose for the three different types of investigated samples Note that for PDF data no error is provided and therefore also the lattice expansion calculated from the PDF fits does not possess an uncertainty. Therefore, the error propagation of the shift from the (440) reflex is provided. See the supporting information for details on how the tabulated values were determined.

Sample	Shift (440) (%)	Expansion in *a* (%)	Radiation dose (kGy)
ID31-1-EtOH–H_2_O	−0.36 ± 0.06	0.34	40000
ID31-2-EtOH–H_2_O	−0.53 ± 0.05	0.47	40000
ID31-1-H_2_O	−0.00 ± 0.03	0.00	40000
ID31-2-H_2_O	0.00 ± 0.07	0.00	40000
ID15-A-1-EtOH–H_2_O	−0.13 ± 0.06	†	195–390
ID15-A-2-EtOH–H_2_O	−0.02 ± 0.04	0.00	195–390

† Data quality for the PDF fit too bad.
