# Supplementary material for: Beam-induced redox chemistry in iron oxide nanoparticle dispersions at ESRF–EBS
Source: J Synchrotron Radiat. 2023 Jan 13;30(Pt 2):440–4. doi: 10.1107/S1600577522011523 (PMC10000811; doi:10.1107/S1600577522011523)
Supplement: Supplementary file 1 [file s-30-00440-sup1.pdf]

JOURNAL OF SYNCHROTRON RADIATION

Volume 30 (2023)

Supporting information for article:

Beam-induced redox chemistry in iron oxide nanoparticle dispersions at ESRF-EBS

Sabrina L. J. Thomä and Mirijam Zobel

S1. Sample preparation

The IONPs with an approximate size of 7 nm or 15 nm were obtained as reported in (Thomä *et al.*, 2019) from hydrolysis of iron chelated complexes in diethylene glycol (DEG) or DEG/N-methyl-2,2‘-iminodiethanol (NMDEA) mixture (70:30 wt%, only ID31-2) according to (Caruntu *et al.*, 2004, Qu *et al.*, 2011). 1 mmol $FeCl_2 \cdot 4\ H_2O$ (199 g/mol) and 2 mmol $FeCl_3 \cdot 6\ H_2O$ (270 g/mol) were dissolved in 40 g DEG (DEG/NMDEA). A solution of 8 mmol NaOH (40 g/mol) in 20 g DEG (DEG/NMDEA) was added. The reaction solution was then degassed under argon for about 2 hours and subsequently heated to 220°C with a heating rate of 130°C/h. The temperature was kept constant for 2 hours and then cooled to about 120°C. At this point the respective capping ligand dissolved in a DEG:H_2O mixture was added (see Table S1). After the addition of the capping-agent solutions, the nanoparticle suspensions were stirred for another 10-15 minutes, while cooling to room temperature. When cooled to room temperature or the next day, the nanoparticle powder was precipitated by addition of ca. the double volume of acetone and isolated on a magnet. For purification, the powder was washed with absolute ethanol at least four times. To obtain the EtOH-H_2O dispersions, after the last drying step, the nanoparticle powders was freshly re-dispersed by removing supernatant ethanol, drying at air and then adding water (10 mM HCl in case of ID15-A-2). This gives an aqueous dispersion with a little amount of residual EtOH. The concentration of the dispersions was determined gravimetrically (see Table S1). Dispersions in pure H_2O were received by letting the powders dry completely (weakly covered with tin foil) after the last purification step, in the ventilated air of the fumehood, at least over night. The dry powder was then re-dispersed in water prior to the beamtime with a concentration of 10 g/L.

Table S1 Details on prepared samples like type and amount of capping ligand and concentration of IONPs in EtOH-H_2O dispersions.

Sample	Capping ligand mmol per mmol Fe	concentration g/L
ID31-1-EtOH-H_2O	tri-sodium citrate 0.4	5
ID31-2-EtOH-H_2O	tri-sodium citrate 0.4	2
ID15-A-1-EtOH-H_2O	aminobenzoic acid 1.0	2
ID15-A-2-EtOH-H_2O	tri-sodium citrate 1.0	3

S2. Calculation of radiation dose

The radiation dose after 60 and 250 s (for ID31 and ID15-A) in kGy (Gray, 1 Gy = $\frac{J}{kg}$), respectively, was calculated according to (Bondaz *et al.*, 2020). With the approximation of the sample being water the mass of the sample complies to the irradiated volume. The beam absorption for the respective energies for water and a target length of 1 mm (diameter of capillary) was estimated to be 2% with an

online calculator based on NIST data (https://web-docs.gsi.de/~stoe_exp/web_programs/x_ray_absorption/index.php).

Radiation dose ID31 after EBS-upgrade:

The photon flux on the sample was measured with a diode and was $1 \cdot 10^{14}$ photons per second.

$$dose = \frac{E_{transferred}}{m_{sample}} \cdot time = \frac{65\ keV \cdot 1.6 \cdot 10^{-16} \frac{J}{keV} \cdot 1 \cdot 10^{14} \frac{1}{s} \cdot 0.02}{(0.03 \cdot 0.01 \cdot 0.1) cm^3 \cdot 1 \frac{g}{cm^3} \cdot 10^{-3}} \cdot 60\ s = \frac{0.02 \frac{J}{s}}{3 \cdot 10^{-8} kg} \cdot 60\ s = 40{,}000\ kG$$

Radiation dose ID15-A before EBS-upgrade:

The photon flux on the sample was estimated by the respective beamline scientist according to (Vaughan et al., 2020). The total flux focused on the sample with the used optics ('Transfocators and LLM') at ca 70 keV is now about 10^{12} ph/s (Vaughan *et al.*, 2020, see Figure 6) for full focussing, but ca. 2x higher for the focussing applied in the experiment. Before the EBS upgrade the flux was 20-40 times lower (Vaughan *et al.*, 2020, see Figure 2) and therefore the flux was estimated to be between $5 \cdot 10^{10}$-$1 \cdot 10^{11}$ ph/s. Two limiting values for the radiation dose were calculated.

$$dose = \frac{E_{transferred}}{m_{sample}} \cdot time = \frac{68\ keV \cdot 1.6 \cdot 10^{-16} \frac{J}{keV} \cdot 5 \cdot 10^{10} \frac{1}{s} \cdot 0.02}{(0.012 \cdot 0.012 \cdot 0.1) cm^3 \cdot 1 \frac{g}{cm^3} \cdot 10^{-3}} \cdot 250\ s = \frac{1.09 \cdot 10^{-5} \frac{J}{s}}{1.4 \cdot 10^{-8} kg} \cdot 250\ s = 195\ kG$$

$$dose = \frac{E_{transferred}}{m_{sample}} \cdot time = \frac{68\ keV \cdot 1.6 \cdot 10^{-16} \frac{J}{keV} \cdot 1 \cdot 10^{11} \frac{1}{s} \cdot 0.02}{(0.012 \cdot 0.012 \cdot 0.1) cm^3 \cdot 1 \frac{g}{cm^3} \cdot 10^{-3}} \cdot 250\ s = \frac{2.18 \cdot 10^{-5} \frac{J}{s}}{1.4 \cdot 10^{-8} kg} \cdot 250\ s = 390\ kG$$

S3. Composition and crystal structure of IONPs

The IONPs are phase-pure (no wüstite side phase) and crystallize in inverse spinel structure. In Figure S1 panel a) it can be seen, that the reference pattern for maghemite in $P4_32_12$ structure describes all observed reflexes of the IONPs. (Greaves, 1983) The utilized stoichiometry of the iron precursor salts in the synthesis should result in a Fe_3O_4 (magnetite) stoichiometry. Yet, since the IONP powders are stored in air and they are known to oxidize to γ-Fe_2O_3 (maghemite) over time, we expect a non-stochiometric composition in between Fe_3O_4 and γ-Fe_2O_3. (Cervellino *et. al.*, 2014, Cooper *et al.*, 2020) The presence of ordered vacancies (due to oxidation of Fe^{2+} to Fe^{3+}) is affirmed by the presence (even though quite weakly above background level) of the three reflexes marked with *. Those are only present when the symmetry of cubic spinel structure is reduced to tetragonal by vacancy ordering on octahedral sites. (Greaves, 1983) For one IONP powder a PDF fit with $P4_32_12$ structure (see Figure S1 panel b) was performed and the best fit was indeed found for an occupation of the Fe(4) site in between magnetite (1) and maghemite (0.33). The results of the performed fits are presented in Table S2.

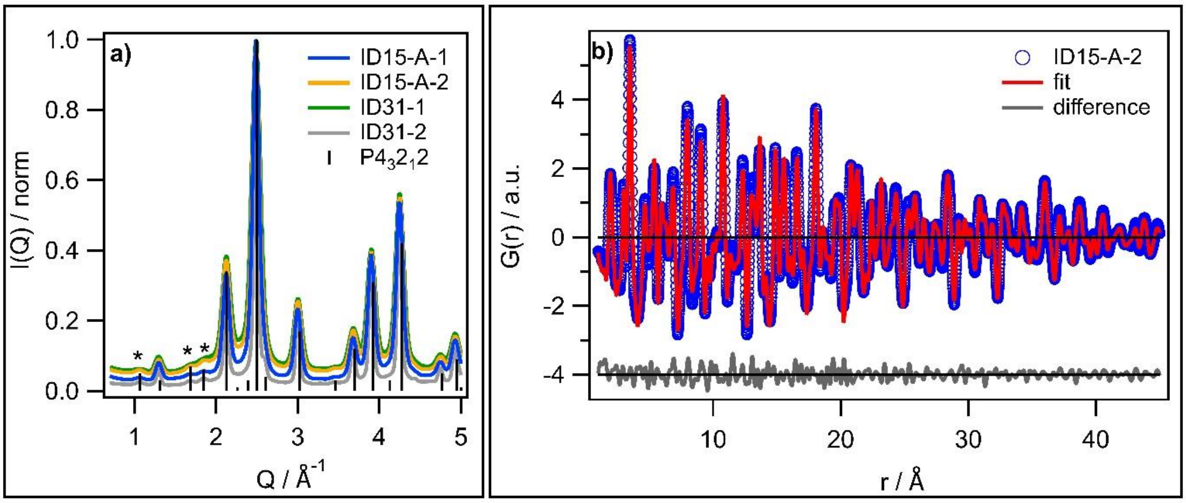

Figure S1 a) XRD patterns of IONP powders of respective samples studied in dispersion. All reflexes can be explained with the maghemite reference pattern in $P4_32_12$ structure. b) Best PDF fit with $P4_32_12$ structure model to IONP powder with an occupancy of the Fe(4) site of 0.53.

Table S2 Overview of results of PDF fits with maghemite/magnetite model in $P4_32_12$ structure with different occupation of Fe(4) site.

parameters	Maghemite composition	Magnetite composition	Occupation Fe(4) free
a (Å)	8.3861	8.3808	8.3851
c (Å)	8.3382	8.3549	8.3423
scale	0.6382	0.6077	0.6278
delta 2 (Å^2)	2.0378	2.5079	2.1904
spherical diameter (Å)	74.4650	70.8543	73.2684
u_{iso} (Fe) (Å^2)	0.0046	0.0046	0.0046
u_{iso} (O) (Å^2)	0.0144	0.0140	0.0144
occupation Fe(4)	0.33 (fixed)	1.00 (fixed)	0.5329
R_w (goodness of fit)	0.150	0.161	0.146

S4. Evaluation of ethanol (EtOH) content

Since the IONPs were freshly re-dispersed in water directly after the purification, the dispersion contains a little amount of residual EtOH. The fact, that it is a low concentrated EtOH-H_2O mixture can be recognized by the FSDP of the IONP dispersion of this mixture being shifted a little to lower *Q*-values in comparison to water and IONPs in water, since pure EtOH exhibits its FSDP at ca. 1.53 Å^{-1} (see Figure S2 panel a)). Further, the FSDP also seems to broaden a bit. To track the EtOH amount

down at ID31, a 6 vol% EtOH-H_2O mixture was measured, too, and the diffraction patterns of IONP dispersion and this mixture match pretty well (see Figure S2 panel b)). Consequently, the amount of EtOH in the IONP dispersions is about 6 vol%. In order to show, that the EtOH amount is similarly low in all evaluated samples before and after the upgrade, the FSDP of the dispersions and water was fitted with a Gaussian fit on a linear background between 1.6-2.4 Å^{-1} (see Figure S2 panel c). The region for the fit wasn't chosen bigger in order to exclude the region of the Bragg peak of IONP dispersions at ca. 2.48 Å^{-1}. From Figure S2 panel a and b it is evident, that this region is sufficient to describe the shift caused by the little amount of contained EtOH. Compared to the position (and FWHM) of pure water, the FSDPs of dispersions are shifted to smaller Q values (and are broader), see Table S3.

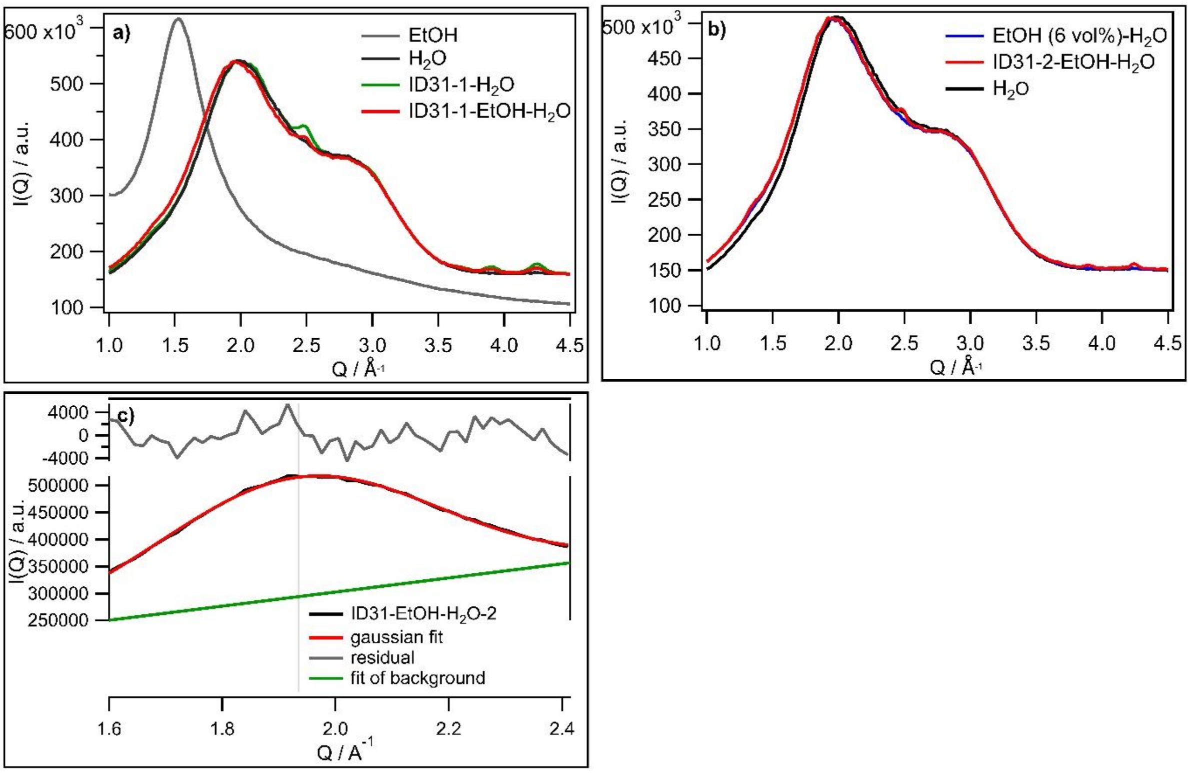

Figure S2 a) Diffraction pattern of an IONP dispersion in EtOH-H_2O mixture in comparison to water, IONPs in water and pure EtOH. b) Diffraction pattern of an IONP dispersion in EtOH-H_2O solution in comparison to a 6 vol% EtOH-H_2O mixture and water. c) Gaussian fit on linear background to the FSDP of one of the IONP dispersion samples.

Table S3 Results of Gaussian fits (location and FWHM) on linear background to FSDP of water and FSDP of IONP dispersions in comparison.

sample	location FSDP [Å^{-1}]	FWHM FSDP [Å^{-1}]
H_2O-ID31	1.9640 ± 0.0018	0.5619 ± 0.0084
EtOH 6vol% -H_2O-ID31	1.9313 ± 0.0027	0.5779 ± 0.0109
ID31-1-EtOH-H_2O	1.9333 ± 0.0021	0.5757 ± 0.0088
ID31-2-EtOH-H_2O	1.9351 ± 0.0025	0.5762 ± 0.0105
H_2O-ID15-A	1.9610 ± 0.0018	0.5639 ± 0.0086
ID15-A-1-EtOH-H_2O	1.9420 ± 0.0022	0.5746 ± 0.0098
ID15A-2-EtOH-H_2O	1.9270 ± 0.0028	0.5832 ± 0.0118

S5. Gaussian fit to (440) reflex

In order to quantify the observed shift in relation to the scan number, for IONP dispersions in low concentrated EtOH-H_2O solution at ID31 after the EBS upgrade, the (440) reflex in the *Q*-range between 4.10 – 4.40 Å^{-1} of three scans (1,3 and 10) was fitted with a Gaussian on a background (see Figure S3). As background function a line was chosen. For comparison, the Gaussian fits were also performed for samples, where no shift of the reflexes was observed (IONPs in H_2O at ID31, IONPs in EtOH-H_2O at ID15-A before EBS upgrade). The results are presented in Table S4. The locations (loc) of (440) listed in Table S4 were taken to calculate the peak shifts in % presented in Table 1 according to $shift = \frac{loc_{Scan10} - loc_{Scan1}}{loc_{Scan10}} \cdot 100$. The error on this shift was determined with a Gaussian error propagation.

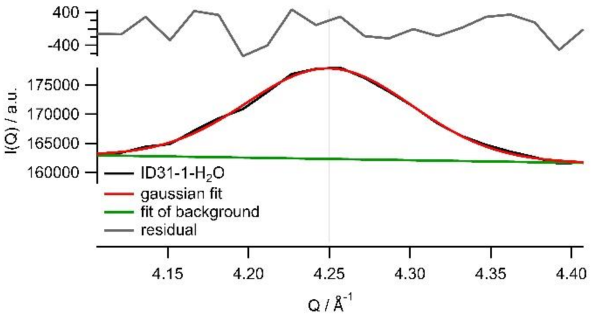

Figure S3 Gaussian fit applied to the (440) reflex in the Q-region between 4.10 – 4.40 Å^{-1}. The background was fitted with a linear slope.

Table S4 Results of Gaussian fits (location and FWHM) of (440) reflex of three scans of each sample.

sample	location (440) [$Å^{-1}$]	FWHM (440) [$Å^{-1}$]
ID31-1-EtOH-H_2O Scan 1	4.2458 ± 0.0014	0.1189 ± 0.0043
ID31-1-EtOH-H_2O Scan 3	4.2271 ± 0.0020	0.1288 ± 0.0066
ID31-1-EtOH-H_2O Scan 10	4.2304 ± 0.0020	0.1293 ± 0.0065
ID31-2-EtOH-H_2O Scan 1	4.2433 ± 0.0014	0.0627 ± 0.0037
ID31-2-EtOH-H_2O Scan 3	4.2345 ± 0.0015	0.0668 ± 0.0041
ID31-2-EtOH-H_2O Scan 10	4.2208 ± 0.0016	0.0731 ± 0.0044
ID31-1-H_2O Scan 1	4.2499 ± 0.0008	0.1203 ± 0.0026
ID31-1-H_2O Scan 3	4.2491 ± 0.0009	0.1209 ± 0.0026
ID31-1-H_2O Scan 10	4.2497 ± 0.0009	0.1210 ± 0.0027
ID31-2-H_2O Scan 1	4.2482 ± 0.0020	0.0636 ± 0.0051
ID31-2-H_2O Scan 3	4.2483 ± 0.0021	0.0635 ± 0.0055
ID31-2-H_2O Scan 10	4.2483 ± 0.0023	0.0643 ± 0.0059
ID15-A-1-EtOH-H_2O Scan 1	4.2453 ± 0.0017	0.0839 ± 0.0044
ID15-A-1-EtOH-H_2O Scan 3	4.2425 ± 0.0019	0.0818 ± 0.0019
ID15-A-1-EtOH-H_2O Scan 10	4.2396 ± 0.0021	0.0844 ± 0.0056
ID15-A-2-EtOH-H_2O Scan 1	4.2649 ± 0.0011	0.0876 ± 0.0030
ID15-A-2-EtOH-H_2O Scan 3	4.2649 ± 0.0011	0.0867 ± 0.0031
ID15-A-2-EtOH-H_2O Scan 10	4.2642 ± 0.0012	0.0859 ± 0.0032

S6. PDF modelling for lattice parameter

In order to show, that the shift to lower Q-values in $I(Q)$, is a global phenomenon concerning the whole diffraction pattern and is indeed related to lattice expansion, the data was also evaluated in real space. d-PDFs (difference pair distribution functions; dispersion data minus water background) were evaluated for three scans (1,3 and 10) of each sample. Figure S4a) illustrates that the distance correlations in these d-PDFs of an IONP dispersion in EtOH-H_2O solution in the mid-r range are shifted to higher r with increasing scan number indicating lattice expansion. These d-PDFs were fitted in the range from 15-100 Å in order to quantify the lattice expansion (see Figure S4b) for one exemplary fit. Note, that the low r-range could not be fitted, since it contains signal from residual EtOH. For simplicity (no averaging

of lattice parameters, in order to see lattice expansion) the cubic Fd$\bar{3}$m model, in which magnetite/maghemite can also be described, was taken for the fitting. Fitted values were the unit cell parameter (a=b=c), a scale factor to match experimental and theoretical intensity, the spherical diameter, u_{iso} of iron and oxygen – isotropic atomic displacement parameters, as well as the oxygen position. In Table S5 the obtained lattice parameters as well as the goodness of fit parameters are listed for all evaluated samples. The lattice expansion (lattice exp) in % presented in Table 1 was calculated with the presented values in Table S5 according to $lattice\ \exp = \frac{a_{Scan10} - a_{Scan1}}{a_{Scan10}} \cdot 100$. Since the fitted PDF data does not possess an error, the error provided by the fits in diffpy-cmi are invalid and an error propagation was not performed.

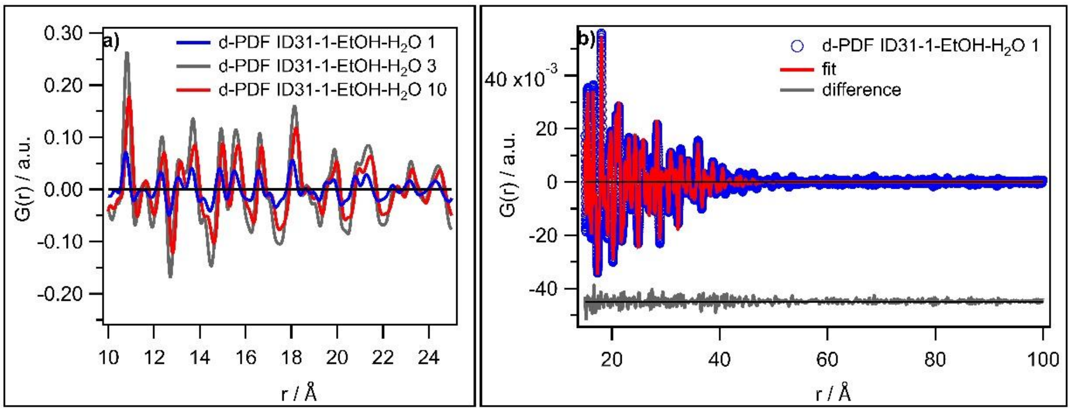

Figure S4 a) For the mid-*r* range the distance correlations in the d-PDF of IONP dispersions (minus water background) in low concentrated EtOH-H_2O solution are shifted to higher *r* with increasing scan number. This indicates lattice expansion. b) Exemplary fit to the d-PDF of an IONP dispersion between 15-100 Å in order to quantify the lattice expansion.

Table S5 Lattice and goodness of fit parameters obtained from PDF fits to d-PDFs of three scans (1,3, and 10) of the evaluated samples. The fit quality for fits to d-PDFs of sample ID-15-A-1-EtOH-H_2O were too low to give conclusive results.

sample	lattice parameter a=b=c [Å]	Rw (goodness of fit)
ID31-1-EtOH-H_2O Scan 1	8.3715	0.163
ID31-1-EtOH-H_2O Scan 3	8.4098	0.218
ID31-1-EtOH-H_2O Scan 10	8.3998	0.212
ID31-2-EtOH-H_2O Scan 1	8.3753	0.359
ID31-2-EtOH-H_2O Scan 3	8.3946	0.385
ID31-2-EtOH-H_2O Scan 10	8.4148	0.452
ID31-1-H_2O Scan 1	8.3647	0.165
ID31-1-H_2O Scan 3	8.3659	0.161
ID31-1-H_2O Scan 10	8.3647	0.164
ID31-2-H_2O Scan 1	8.3668	0.397
ID31-2-H_2O Scan 3	8.3670	0.411
ID31-2-H_2O Scan 10	8.3668	0.436
ID15-A-2-EtOH-H_2O Scan 1	8.3775	0.531
ID15-A-2-EtOH-H_2O Scan 3	8.3761	0.630
ID15-A-2-EtOH-H_2O Scan 10	8.3773	0.592

References

Bondaz, L., Fontaine, P., Muller, F., Pantoustier, N., Perrin, P., Morfin, I., Goldmann, M. & Cousin, F. (2020). Langmuir 36, 6132-6144.

Caruntu, D., Caruntu, G., Chen, Y., O'Connor, C. J., Goloverda, G. & Kolesnichenko, V. L. (2004). *Chem Mater* **16**, 5527-5534.

Cervellino, A., Frison, R., Cernuto, G., Guagliardi, A. & Masciocchi, N. (2014). Journal of Applied Crystallography 47, 1755-1761.

Cooper, S. R., Candler, R. O., Cosby, A. G., Johnson, D. W., Jensen, K. M. O. & Hutchison, J. E. (2020). ACS Nano 14, 5480-5490.

Greaves, C. (1983). *Journal of Solid State Chemistry* **49**, 325-333.

https://web-docs.gsi.de/~stoe_exp/web_programs/x_ray_absorption/index.php, access 31.07.2022.

Qu, H., Caruntu, D., Liu, H. & O'Connor, C. J. (2011). *Langmuir* **27**, 2271-2278.

Thomä, S. L. J., Krauss, S. W., Eckardt, M., Chater, P. & Zobel, M. (2019). *Nature Communications* **10**, 995.

Vaughan, G. B. M., Baker, R., Barret, R., Bonnefoy, J., Buslaps, T., Checchia, S., Duran, D., Fihman, F., Got, P., Kieffer, J., Kimber, S. A. J., Martel, K., Morawe, C., Mottin, D., Papillon, E., Petitdemange, S., Vamvakeros, A., Vieux, J. P. & Di Michiel, M. (2020). *J Synchrotron Radiat* **27**, 515-528.
